# Validity and internal consistency of a Hausa version of the Ibadan knee/hip osteoarthritis outcome measure

**DOI:** 10.1186/1477-7525-6-86

**Published:** 2008-10-22

**Authors:** Adesola C Odole, Aderonke O Akinpelu

**Affiliations:** 1 Department of Physiotherapy, College of Medicine, University of Ibadan, Ibadan, Oyo State, Nigeria

## Abstract

**Background:**

The Ibadan Knee/Hip Osteoarthritis Outcome Measure (IKHOAM) was developed for measuring end results of care in patients with knee or hip OA in Nigeria. The purpose of this study was to validate a Hausa translation of IKHOAM in order to promote its use among the Hausa populations of Nigeria and other West African countries.

**Methods:**

Sixty-seven patients with knee OA, literate in Hausa and English, recruited consecutively from all government hospitals in Kano were assessed on both English and Hausa versions of IKHOAM. The order of assessment with the versions was randomized and separated by 24 hours. Participants also rated their pain intensity on the Visual Analogue Scale. Data was analyzed using the Spearman Rank Order correlation and Cronbach's alpha.

**Results:**

The participants (17 males, 50 females) were aged 55.7 ± 13.4 years. Participants' scores on the Hausa version correlated significantly with the original version (r = 0.67, p = 0.000) and with pain intensity scores on the Visual Analogue Scale (r = -0.24, p = 0.005). The Cronbach's alpha for correlation on the different parts of the Hausa version ranged between 0.28 and 0.95.

**Conclusion:**

The Hausa version of IKHOAM meets the criteria for validity and internal consistency and may be used in the Hausa speaking parts of Nigeria and other West African countries.

## Background

The Ibadan Knee/Hip Osteoarthritis Outcome Measure (IKHOAM), a Nigerian culture and environment-friendly clinical tool was developed at the University of Ibadan, Nigeria for measuring end results of care in patients with knee or Hip OA [1]. The tool was made specific to Knee/Hip joints because among Nigerian patients, the knee is the most frequently affected by OA followed by the hip [2,3]. It is a 3 domain, 33-item clinical instrument. Parts1 and 2 of IKHOAM are patient-report. Part 1 measures the degree of limitations and nature of assistance required in 25 relevant activities of daily living on a five (0–4) point ordinal scale. Part 2 measures the degree of participation restriction in 3 activities on a four (0–3) point ordinal scale. Part 3 comprises 5 physical performance tests, which is rated by the clinician on five and six point ordinal scales. IKHOAM has been shown to demonstrate initial criteria towards validity and responsiveness [1].

Nigeria is a multi-ethnic country with over 500 indigenous languages. The three major Nigerian indigenous languages are Hausa, Igbo and Yoruba [4]. Probably for ease of communication among the various ethnic groups in Nigeria, the official language of communication is English (the language of the country's former colonial master). The original language of IKHOAM is therefore English. It has however been reported that a sizeable number of patients in Nigeria do not speak or write English [5]. We therefore recognized the need to translate IKHOAM into the 3 major indigenous languages of Nigeria in order to facilitate its use among this group of patients. In an earlier study, the Yoruba version of IKHOAM has been shown to be valid and internally consistent [6]. The purpose of this study was to translate IKHOAM into Hausa language and to investigate its validity and internal consistency. This would hopefully promote the use of IKHOAM in Nigeria and other West African countries where Hausa language is spoken.

We hypothesized that there would be significant correlation between the participants' scores on the Hausa and English versions of IKHOAM (cross-sectional construct validity) as well as between the Hausa version of IKHOAM and pain intensity scores (divergent validity). We also postulated that the correlations among the 3 parts of the Hausa version of IKHOAM would be significant (internal consistency).

## Methods

We followed the recommended guidelines for the process of translation of self-report measures by Beaton et al [7] to translate IKHOAM into the Hausa language. Two linguists proficient in both English and Hausa Languages, whose mother tongue is Hausa independently translated the English version of IKHOAM (see Additional file 1) in to Hausa and then developed a reconciled version. The reconciled version was then back translated into English language by a third linguist who was not associated with the initial translation.

A professional expert group, composed of two of the developers of IKHOAM, one of the translators, and a Physiotherapist, whose mother tongue is Hausa, and who is fluent in both English and Hausa languages revised the back-translation. Five patients with knee OA were asked to complete parts 1 and 2 of the consensus Hausa translated version of IKHOAM and they were rated on the physical tests (part 3) by another physiotherapist, fluent in Hausa language. The patients subsequently participated in a cognitive debriefing interview. All the 5 patients reported clarity of the Hausa language and ease of understanding of all the items. The final version of the Hausa translation of IKHOAM (see Additional file 2). The anchors (English) on the visual analogue scale were also translated into Hausa language through a forward-back translation process (see Additional file 3)

### Investigation on Validity and Internal Consistency

Participants were 67 patients diagnosed with Knee OA grade 3 or less according to Kellgren and Lawrence grading system, who were bilingual in English and Hausa languages. Patients with obvious or documented evidence of cardiovascular disease or concurrent neuromuscular and musculoskeletal diseases and those who had previous surgeries to the knee and or hip were excluded from the study. Hausa language is the first language (mother tongue) of the 67 patients. They were recruited from 3 government hospitals (25 participants from an orthopaedic hospital, 31 from a university teaching hospital and11 from a state hospital) in Kano, Northern Nigeria. The procedure was explained to each participant and his/her informed consent (verbally and written) was obtained. Socio demographic data (age, sex, marital status) and clinical history of OA were obtained through interview and from hospital files.

Participants were assessed using both the English and the Hausa versions of IKHOAM through an interview conducted by one of the authors (ACO) on parts 1 and 2 (patients' self-report) while part 3 (clinician-measured part) was measured by same person. The order of assessment using both versions of IKHOAM was randomized using the fish-bowl technique. Participants were also assessed on the Visual Analogue Scale (VAS) for pain intensity. This was to investigate the divergent validity of Hausa version of IKHOAM since most activity limitations in OA are consequent to pain. The VAS has been validated in the Nigerian clinical setting [8,9].

### Data Analysis

Descriptive statistics of mean and standard deviation were used to summarize data. Gender, marital status, age ranges of participants, duration of onset of OA and joints affected were summarized with proportions. Participants' scores obtained on the Hausa and English versions of IKHOAM were subjected to Spearman rank order correlation to demonstrate cross-sectional construct validity of the Hausa version of IKHOAM. The divergent validity of the Hausa version of IKHOAM was analyzed by subjecting participants' scores on the Visual Analogue Scale and the Hausa version of IKHOAM to Spearman rank Order correlation. Internal consistency of the 3 parts of the Hausa version of IKHOAM was calculated using the Cronbach's alpha. Level of significance was set at 0.05. The SPSS 12 software program was used in data analysis [10].

## Results

**Figure 1:**
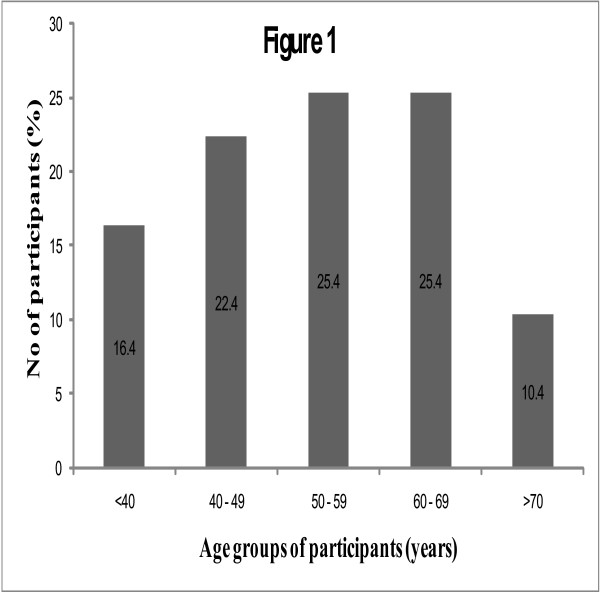
Distribution of onset of OA.

**Figure 2:**
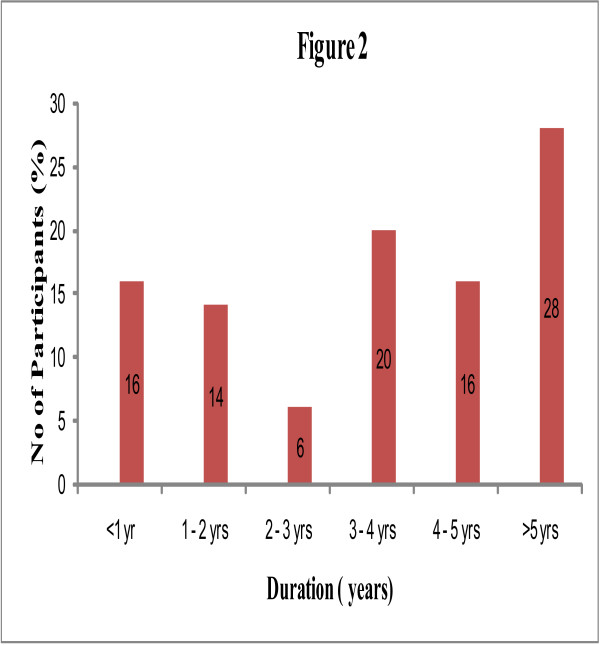
Age distribution of participants.

The participants were aged 55.7 ± 13.4 years. Seventeen [25.4%] were males and 50 (74.6%) were females. The mean age of the males was 55.3 ± 8.4 years and that of the females was 55.6 ± 12.0 years [Table 1]. The majority of the participants [61.2%] were within the age range of 50–69 years [Figure 1]. Fifty-five [82%] participants were married, 5 [7.5%] were widowed, 5 [8%] were divorced and 2 [3%] were single. The distribution of onset of OA is presented in Figure 2. Fifty [74.6%] had OA of one or both knee joints, 15 [22.4%] had affectation of one or both hip joints and 2 [3%] had involvement of both hips and both knees.

**Table 1:** Summary of participants' data

Characteristics	$\bar{\text{X}}$ (mean)	S.D
**Gender**		
Male (17)	55.3	8.4
Female (50)	55.6	12.0
Total (67)	55.7	13.4
		
**IKHOAM Scores**		
English	82.16	14.58
Hausa	84.81	15.18
		
**Pain Intensity Scores**	4.76	1.60

### Validity

The mean score of the participants on the English version of IKHOAM was 82.16 ± 14.58 and their mean score on the Hausa version of IKHOAM was 84.81 ± 15.18 [Table 1]. The mean pain intensity score of participants was 4.76 ± 1.60 [Table 1]. The mean IKHOAM score on the English version correlated significantly with the mean of the Hausa translated version (r = 0.67. p = 0.000) [Table 2]. The mean pain intensity score correlated negatively and significantly with the mean IKHOAM scores on the Hausa translated version (r = -0.24, p = 0.05) [Table 2].

**Table 2:** Spearman's rank order correlation coefficients between scores on English and Hausa versions of IKHOAM and the visual analogue scale

	IKHOAM Scores (English)	Pain Intensity Score
IKHOAM Score (Hausa)	0.67*	-0.24**

* P = 0.000

** P = 0.005

### Internal consistency

There was a positive significant correlation between the patient- measured parts (parts 1 and 2) and clinician-measured part [parts 3] (α = 0.73, p = 0.000) [Table 3]. There was a positive significant correlation between part 1 and part 3 (α = 0.49, p = 0.005) and between part 2 and part 3 (α = 0.65, p = 0.000). The correlation between part 1 and part 2 (α = 0.28, p = 0.005) was positive and significantly significant though low. There was significant correlations between the total scores on all the three parts and each of the three parts (α = 0.64 for part 1, 0.84 for part 2, 0.92 for part 3) [Table 3]. There was a positive significant correlation between the patient measured parts (parts 1 and 2) and the total scores on all the three parts (α = 0.95; Table 3).

**Table 3:** Cronbach's alpha for the different parts of Hausa version of IKHOAM

	Total (Parts 1,2 & 3)	Part 1	Part 2	Part 3
Part 1	0.64*			
Part 2	0.84*	0.28**		
Part 3	0.92*	0.49**	0.65*	
Part 1 & 2	0.95*			0.73*

* P = 0.000

** P = 0.005

## Discussion

During the process of translating the English version of IKHOAM into Hausa, the meanings of all items were retained in the back translation of the reconciled Hausa version and all the patients involved in the cognitive debriefing interview reported no difficulty in clarity of the language and ease of understanding of all the items. This is probably because there was no cross-cultural adaptation per se, although we followed the guidelines for cross-cultural adaptation by Beaton et al [7]. IKHOAM was only translated into another language within the same cultural context. This observation supports the fact that IKHOAM is a Nigerian culture and environment-friendly clinical instrument.

The female to male ratios of 3:1 supports the fact that in hospital based studies, knee/hip OA is more common in Nigerian females than males [11,12,2] and could be a reflection of what obtains in the overall population of OA patients of moderate female bias [11]. The fact that majority (61.2%) of all the patients with Knee/Hip Osteoarthritis in the study was aged 50 years and above with mean age of 55.7 ± 13.4 years supports the fact that OA may be regarded as a disease of middle and old age.

The scores obtained on the Hausa version correlated significantly with those on the English version. It implies that the Hausa version measures the same construct as the English version. The correlation coefficient of 0.67 between the Hausa and English versions found in this study falls within acceptable values (0.60 – 0.80) for construct validity [13]. The absence of data on the pain duration of the participants in this study is a limitation of this study as the chronicity of their pain could not be ascertained. The significant correlation between IKHOAM scores on the Hausa version and pain intensity scores (r = -0.24) provides the evidence that the Hausa version demonstrates initial criterion for divergent validity. It is not surprising that this correlation coefficient is low, since the IKHOAM and the VAS measure dissimilar constructs. Values of correlation coefficient between dissimilar constructs usually fall between 0.20 and 0.60 [14]. The results of this study support that of Dawson et al (2005). In that study, divergent construct validity was supported by the correlation (r = 0.34) between pain severity and physical function. Several studies comparing dissimilar constructs also fell within this acceptable range [14,15]. The results on divergent validity of Hausa IKHOAM with the use of VAS in this study is a limitation of the study since IKHOAM is multidimensional while VAS has only one item that assesses pain. However, further studies should be carried out to further demonstrate evidence of divergent validity by comparing IKHOAM with measures of different construct e.g. Health Assessment Questionnaire (HAQ), Sickness Impact Profile (SIP).

The Cronbach's alpha values between the different parts (parts 1 and 2; parts 1 and 3; parts 2 and 3; parts 1 & 2 together and part 3) on the Hausa version of IKHOAM indicate that the Hausa version is internally consistent though there is a weak correlation between parts 1 and 2. The Cronbach's alpha of the three parts of the Hausa version ranged between 0.28 and 0.95. These values are comparable to the values got in several studies on validity of different versions of some outcome measures [6,13,16,15]. The significant correlation between the patient's measured part (parts 1 & 2) and the clinician measured part (part 3) on the Hausa version of IKHOAM indicates that changes in functional ability of patients following intervention can be assessed by either the patient's self report or the clinician measure. This is similar to the findings of previous studies on the original (English) version [1] and the Yoruba version [6] that the versions of IKHOAM possess adequate criteria for internal consistency. However, we observed that the correlation between part I (Disability attributes) and part 2 (participation restriction attributes) was lower (α = 0.28) than Cronbach's alpha between other parts of the tool. This may be explained by the fact that many female participants in this study were in purdah, a common cultural/religious practice in the Northern part of Nigeria. Women in purdah have limited social life because they are compelled to stay at home most of the time.

## Conclusion

The Hausa version of IKHOAM is a valid and internally consistent translation of the English (original) version. It may be used to assess outcomes of care in patients with knee or hip osteoarthritis in the Hausa-speaking populations of Nigeria and other West African countries. Further studies should be carried out to strongly demonstrate its validity and reliability.

Ethical approval: The joint University of Ibadan and University College Hospital Institutional Review Committee. Protocol number UI/IRC/04/0087.

## Competing interests

The authors declare that they have no competing interests.

## Authors' contributions

AOA conceptualized the study and revised the drafted manuscript. ACO was involved in data acquisition, analysis and interpretation of data and drafting of the manuscript. Both authors participated in the design of the study, read and approved the final manuscript.

## Supplementary Material

Additional file 1 **Ibadan Knee/Hip Osteoarthritis Outcome Measure (IKHOAM).** The data provided the English version of the Ibadan Knee/Hip Osteoarthritis Outcome Measure (IKHOAM). Click here for file

Additional file 2 **Ibadan Knee/Hip Osteoarthritis Outcome Measure (IKHOAM) Hausa version.** The data provided the Hausa version of the Ibadan Knee/Hip Osteoarthritis Outcome Measure (Hausa IKHOAM). Click here for file

Additional file 3 **The English and Hausa versions of the visual analogue scale.** The data provided the English and Hausa versions of the visual analogue scale. Click here for file
