# Supplementary material for: Validity and internal consistency of a Hausa version of the Ibadan knee/hip osteoarthritis outcome measure
Source: Health Qual Life Outcomes. 2008 Oct 22;6:86. doi: 10.1186/1477-7525-6-86 (PMC2582225; doi:10.1186/1477-7525-6-86)
Supplement: Additional file 1 — Ibadan Knee/Hip Osteoarthritis Outcome Measure (IKHOAM). The data provided the English version of the Ibadan Knee/Hip Osteoarthritis Outcome Measure (IKHOAM). [file 1477-7525-6-86-S1.doc]

# Additional file 1

# IBADAN KNEE/HIP OSTEOARTHRITIS OUTCOME MEASURE (IKHOAM)

# Part I: Indicate the extent of limitations you experience in carrying out the following activities using these scales:

**Degree of difficulty:** 4 = no difficulty; 3 = mild difficulty; 2 = moderate difficulty; 1 = severe difficulty; 0 = inability to carry out the activity.

**Nature of Assistance:** 4 = requires no assistance; 3 = requires use of aid(s)/device(s) only; 2 = requires assistance of

one person only; 1 = requires assistance of one person and the use of aid(s); 0 - unable to perform the activity.

|  | Difficulty | Assistance |
| --- | --- | --- |
| 1. Washing all body parts during shower. |  |  |
| 2. Walking within the house |  |  |
| 3. Sweeping with a short broom |  |  |
| 4. Walking outside the house for 15-20 minutes |  |  |
| 5. Putting on under clothes |  |  |
| 6. Getting in and out of a salon car |  |  |
| 7. Hand washing of clothes at floor/low level |  |  |
| 8. Rising from bed/mat |  |  |
| 9. Rising from high chair (dining/office chair) |  |  |
| 10. Putting on/lacing shoes or buckling sandals |  |  |
| 11. Rising from an easy chair or sofa |  |  |
| 12. Sweeping with a long brush/broom or using mop stick |  |  |
| 13. Participating in coitus |  |  |
| 14. Incomplete kneeling/prostrating to show courtesy/greet elders |  |  |
| 15. Getting on/off water closet toilet |  |  |
| 16. Getting in and out of a bus/high vehicle |  |  |
| 17. Standing for at least 15 minutes (waiting at bus stop/working in a  modern kitchen with high cooker) |  |  |
| 18. Manual grass cutting/hoeing/gardening |  |  |
| 19. Sitting on the heels (Islamic praying posture) |  |  |
| 20. Climbing stairs |  |  |
| 21. Picking things from floor/low level (kitchen cabinet) |  |  |
| 22. Kneeling (Christian praying posture) |  |  |
| 23. Sitting on a very low stool (e.g. when cooking on a low stove or  firewood at floor level) |  |  |
| 24. Descending stairs |  |  |
| 25. Using pit/Asiatic toilet |  |  |

Part II: Indicate the extent of restriction you experience participating in the following life situations using the scale below Extent of restriction: 3-full participation, 2- at risk full participation, 1-participation with restriction, 0-no participation

|  | Extent |
| --- | --- |
| 1. Performing duties at work (office or at home. |  |
| **2.**  Travelling for one hour or more |  |
| **3.** Participation in social gatherings (e.g. wedding, naming, funeral,  birthday parties) |  |

Part III: Physical performance tests

**250m Walk Test**

5 - able to walk 250m or more at one stretch

4 - able to walk 200-<250m at one stretch

3 - able to walk 150-<200m at one stretch

2 - able to walk 100-<150m at one stretch

1 - able to walk 50-<100m at one stretch

0 - able to walk <50m at one stretch

**Squat Test**

4 - ≥100^0^ knee flexion

3 - 70-99^0^ knee flexion

2 - 40-69^0^ knee flexion

1 - 10-39^0^ knee flexion

0 - < 10^0^ knee flexion

**One leg Stance Test**

5 - can be maintained for 4 minutes or more

4 - can be maintained for 3-<4 minutes

3 - can be maintained for 2-<3 minutes

2 - can be maintained for 1-<2 minutes

1 - can be maintained for <1 minute

0 - unable to perform the test

**Stairs Climbing Test**

4 - can climb with no difficulty and no assistance

3 - can climb with mild difficulty (one railing)

2 - can climb with moderate difficulty (two railings)

1 - can climb with severe difficulty (two railings and help)

0 - unable to climb.

**Balance Test on a Balance Board**

5 - can balance for 45 seconds or more

4 - can balance for 30-<45 seconds

3 - can balance for 20-<30 seconds

2 - can balance for 10-<20 seconds

1 - can balance for <10 second

0 - unable to balance at all.
