# Supplementary material for: Validity and internal consistency of a Hausa version of the Ibadan knee/hip osteoarthritis outcome measure
Source: Health Qual Life Outcomes. 2008 Oct 22;6:86. doi: 10.1186/1477-7525-6-86 (PMC2582225; doi:10.1186/1477-7525-6-86)
Supplement: Additional file 2 — Ibadan Knee/Hip Osteoarthritis Outcome Measure (IKHOAM) Hausa version. The data provided the Hausa version of the Ibadan Knee/Hip Osteoarthritis Outcome Measure (Hausa IKHOAM). [file 1477-7525-6-86-S2.doc]

**Additional file 2**

# MA’AUNIN SAKAMAKON BINCIKEN CIWO SANYIN KASHI NA GWIWA DA KWATANGWALO NA IBADAN

# Karchi na farko: Nuna matsalolin da kake fuskanta wajen aiwatar da wadanna aiyuka masu zuwa ta hanyar amfani da ma’aunai kamar haka:

**Matsayin wahala:** 4 = babu wahala; 3 = ‘yar wahala; 2 = matsakaiciyar wahala; 1 = matsananciyar wahala; 0 = kasa aiwatar da aikin.

**Yanayin taimako:** 4 = ba a bukatar taimako; 3 = bukatuwa ga amfani da kayan aiki kawai; 2 = bukatuwa ga taimakon mutum daya; 1 = bukatuwa ga taimakon mutum daya da amfani da kayan aiki; 0 – baza a iya aikin ba.

|  | Matsayi | Taimako |
| --- | --- | --- |
| 1. Wanke dukkan bangarorin jiki lokacin wanka |  |  |
| 2. Tafiya a cikin gida |  |  |
| 3. Shara da gajeriyar tsintsiya |  |  |
| 4. Tafiya a wajen gida na tsawon minti 15 – 20 |  |  |
| 5. Sanya tufafin ciki |  |  |
| 6. Hawa ko sauka daga karamar mota |  |  |
| 7. Wanke - wanke /wankin tufafi a tsaye wajen famfo ko daidai misalin  Tebur |  |  |
| 8. Tasowa daga gado/ tabarma |  |  |
| 9. Tasowa daga kujera mai tsawo |  |  |
| 10. Sanya takalmi mai igiya |  |  |
| 11. Tasowa daga kujera ta zamani |  |  |
| 12 Shara da togon burushi ko tsinstiya |  |  |
| 13. Halin yanayin jiki yayin jima’i |  |  |
| 14. Lankwas gwiwa da kwatangwalo a halin tsayuwa domin girmamawa  ko gaida na gaba |  |  |
| 15. Zama akan/tasowa daga masai na zamani |  |  |
| 16. Hawa ko sauka daga babbar mota |  |  |
| 17. Tsayuwa t akalla minti 15 |  |  |
| 18. Shema/Noma ta hanyar amfani da kayan aiki na gargajiya (misali yayin dafa abinci a risho na ajiya a kasa ko yin amfani da icce a kasa) |  |  |
| 19. Zaman salla |  |  |
| 20. Hawa matakala |  |  |
| 21. Dukawa don duako abu daga ma’ajiyin dakin girki ko firiji |  |  |
| 22. Tsugunawa (irin yaddakirista ke yi wajen addu’a) |  |  |
| 23. Zama a gajeruwar kujera |  |  |
| 24. Hawa ko sauka daga matakalar bene |  |  |
| 25. Amfani da masai na gargajiya |  |  |

Kachi na biyu: Nua matsayin waha’ar da kake sha a lokacin da kake wandannan al’amuran rayuwa:

**Matsayin wahalar:** 3-cikakken iyawa, 2- hatsari wajen iyawa, 1-amma da wahala, 0-rshin iyawa

|  | Matsay |
| --- | --- |
| **1. Yin aikace** – aikacen ofis ko na gida |  |
| **2.**  **Motsa jiki da Shakatawa**: Shiga harkokin motsa jiki da  shakatawa kamar wasan dara, ludo, kwallon tebur, kwallon sanda,  tafiya, sassarfa, ninkaya. Kamar |  |
| **3.** Harkokin Zamantakewa: Shiga taron Jama’a ko na addini, wato  masallaci ko coci, daurin aure, radin suna, jana’iza, da bikin  murmar zagayowar ranar haihuwa |  |

Kashi na uku: Gwajin Aiki a Aikace

**Gwajin Tafiya na mita 250.**

5 - Iya tafiyar 250 ko fiye da haka a lokaci daya

4 - Iya tafiya kasa da mita 250zuwa mita 200 a lokaci daya

3 - Iya tafiya kasa da mita 250zuwa mita 150 a lokaci daya

2 - Iya tafiya kasa da mita 150zuwa mita 100 a lokaci daya

1 - Iya tafiya kasa da mita 100zuwa mita 50 a lokaci daya

0 - Iya tafiya kasa da mita 50 a lokaci daya

**Gwaji na Tsugune**

4 - Sama da daraja 100 na lankwasa gwiwa

3 - Daraja 70 zuwa 99 na lankwasa gwiwa

2 - Daraja 40 zuwa 69 na lankwasa gwiwa

1 - Daraja 10 zuwa 39 na lankwasa gwiwa

0 - Knasa da daraja 10 na lankwasa gwiwa

**Gwaji na Tsayawa akan Kafa Daya**

5 - za’a iya yinta na tsawon minti 4 zuwa fiye da haka

4 - za’a iya yinta na kasa da minti 4 zuwa 3

3 - za’a iya yinta na kasa da minti 3 zuwa 2

2 - za’a iya yinta na kasa da minti 2 zuwa 1

1 - za’a iya yinta na kasa da minti 1

0 - ba za’a iya yin wanna gwaji ba.

**Gwajin Hawa Matakalar Bene**

4 - za’a iya hawa ba tare da wata matsala ko taimako ba

3 - za’a iya hawa jerin matakala daya tare da ‘yar karamar wahala

2 - za’a iya hawa jerin matakala biyu tare da matsakaiciyar wahala

1 - za’a iya hawa jerin matakala biyu tare da matsanciyar wahala

0 - ba za’a iya hawa ba.

**Gwaji na Tsayuwa akan Ma’aunin Daidaituwa**

5 - za’a iya daidatuwa na tsawon dakika 45 ko sama da haka

4 - za’a iya daidatuwa na tsawon kasa da dakika 45 zuwa 30

3 - za’a iya daidatuwa na tsawon kasa da dakika 30 zuwa 20

2 - za’a iya daidatuwa na kasa da dakika 20 zuwa 10

1 - za’a iya daidatuwa na tsawon dakika 10.

0 - ba za’a iya daidatuwa ba gada daya.
