# Supplementary material for: Validity and internal consistency of a Hausa version of the Ibadan knee/hip osteoarthritis outcome measure
Source: Health Qual Life Outcomes. 2008 Oct 22;6:86. doi: 10.1186/1477-7525-6-86 (PMC2582225; doi:10.1186/1477-7525-6-86)
Supplement: Additional file 3 — The English and Hausa versions of the visual analogue scale. The data provided the English and Hausa versions of the visual analogue scale. [file 1477-7525-6-86-S3.doc]

**Additional file 3**

## VISUAL ANALOGUE SCALE

ENGLISH

_______________________________________________________

No Pain Worst pain I ever felt

HAUSA

______________________________________________________

Babu radadi Mafi tsananin radadi da nataba ji
